# Supplementary material for: Content-rich biological network constructed by mining PubMed abstracts
Source: BMC Bioinformatics. 2004 Oct 8;5:147. doi: 10.1186/1471-2105-5-147 (PMC528731; doi:10.1186/1471-2105-5-147)
Supplement: Additional File 2 — The original results of the above study (non-essential files are deleted to keep the file size under the limit set by BMC bioinformatics). [file 1471-2105-5-147-S2.bz2 › chilibotAdditionalFile2/dip50/607ID9786864E10989/html/LEP_LEPR.html]

 


 **LEP** and **LEPR** 
  
Found 5609 abstracts in PubMed, retrieved 50.  
 

 What does Google say? 
 PDF only 
| .edu only 

---

**Interactive relationship** (e.g. stimulation, inhibition, etc)

**Neutral relationship**- Placental leptin  [ **LEPR** ]   [ **LEP** ]  content was positively correlated with maternal leptin  [ **LEPR** ]   [ **LEP** ]  BMI ratio r = 0.62 and the resistance index of the umbilical artery r = 0.60.  Ref: 12865594 Gynecol Obstet Invest, 2003
- CONCLUSIONS Regulatory effects of leptin  [ **LEPR** ]   [ **LEP** ]  on insulin secretion could be biphasic at different concentrations of glucose and leptin  [ **LEPR** ]   [ **LEP** ] .  Ref: 12877797 Chin Med J (Engl), 2003
- Significantly higher serum levels of TG and lower HDL C were observed in obese leptin  [ **LEPR** ]   [ **LEP** ]  resistant group, as compared with those in obese leptin  [ **LEPR** ]   [ **LEP** ]  sensitive group.  Ref: 12880566 Zhonghua Yu Fang Yi Xue Za Zhi, 2003
- These altered DIO leptin  [ **LEPR** ]   [ **LEP** ]  and ghrelin responses were associated with 10% lower leptin  [ **LEPR** ]   [ **LEP** ]  receptor Ob Rb mRNA expression in the arcuate ARC, dorsomedial DMN and ventromedial hypothalamic nuclei and 13% and 15% lower ghrelin receptor GHS R mRNA expression in the ARC and DMN than DR rats.  Ref: 12865257 Am J Physiol Endocrinol MetabAm J Physiol Endocrinol Metab, 2003
- Examination of relationships among plasma leptin  [ **LEPR** ]   [ **LEP** ] , proinflammatory cytokines, and stress related parameters revealed a significant positive correlation between the plasma leptin  [ **LEPR** ]   [ **LEP** ]  level and IL 1beta or IL 6.  Ref: 12880724 Burns, 2003
- Following 3wk on a 31% fat HE diet, male N3 F.DIO rats gained 16 20% more carcass and adipose weight with 98% higher plasma leptin  [ **LEPR** ]   [ **LEP** ]  levels, while F.DIO females gained 36 54% more carcass and adipose weight with 130% higher leptin  [ **LEPR** ]   [ **LEP** ]  levels than comparable F344 rats.  Ref: 12855420 Am J Physiol Regul Integr Comp Physiol< MedlineTA>Am J Physiol Regul Integr Comp Physiol, 2003
- Our results showed that the serum leptin  [ **LEPR** ]   [ **LEP** ]  levels of the bulimic patients were similar to those of the healthy controls, with a positive correlation between leptin  [ **LEPR** ]   [ **LEP** ]  and BMI.  Ref: 12880190 Eat Weight Disord, 2003
- GH is lipolytic and patients with active acromegaly have lowered serum leptin  [ **LEPR** ]   [ **LEP** ]  compared to age, sex and body mass index BMI matched controls, but a direct influence of GH on serum leptin  [ **LEPR** ]   [ **LEP** ]  remains unclear.  Ref: 12864793 Clin Endocrinol (Oxf)Clin Endocrinol (Oxf), 2003
- RESULTS Serum level of leptin  [ **LEPR** ]   [ **LEP** ]  was 2.74 45.12 micro g L and 0.53 10.18 micro g L in obese and normally weighted children, respectively, with an average level of leptin  [ **LEPR** ]   [ **LEP** ]  log significantly higher in obese group than that in control group P <.  Ref: 12880566 Zhonghua Yu Fang Yi Xue Za Zhi, 2003
- The observed effects of inhibitors of transcription on leptin  [ **LEPR** ]   [ **LEP** ]  gene expression and leptin  [ **LEPR** ]   [ **LEP** ]  secretion are consistent with a long term transcriptional mechanism for insulin stimulated glucose metabolism to increase leptin  [ **LEPR** ]   [ **LEP** ]  production.  Ref: 12878197 Biochem Biophys Res CommunBiochem Biophys Res Commun, 2003

**Non-interactive relationship** (e.g. studied together, co-existance, homology, etc.)

- The levels of leptin  [ **LEPR** ]   [ **LEP** ]  a plasma protein synthesised in human adipose tissue are increased in obese subjects and significantly decrease after a major weight loss.  Ref: 12880185 Eat Weight Disord, 2003
- RESULTS Fasting leptin  [ **LEPR** ]   [ **LEP** ]  was significantly higher P& ; & ; #60 ; 0.05 after the High CHO 18.4 2.6 ng ml 1 mean s.e.m. than after both the Low CHO and the High CHO Ex interventions, which did not differ significantly from each other 16.9 2.1 and 15.5 2.0 ng ml 1, respectively.  Ref: 12861230 Int J Obes Relat Metab DisordInt J Obes Relat Metab Disord, 2003
- With the exciting observations that the obese ob gene product leptin  [ **LEPR** ]   [ **LEP** ] , may also modulate neuroendocrine functions, we examined leptin  [ **LEPR** ]   [ **LEP** ]  s ability to prevent the consequences of fasting on reproductive hormones.  Ref: 12856798 Endocr Res, 2003
- This study examines whether leptin  [ **LEPR** ]   [ **LEP** ]  levels, independent of body mass and insulin resistance, are a risk factor for hypertension and left ventricular hypertrophy.METHODS AND PARTICIPANTS A population based, cross sectional sample of 410 adults from rural Spain was studied.  Ref: 12872039 J HypertensJ Hypertens, 2003
- METHODS We studied the effects of exogenous leptin  [ **LEPR** ]   [ **LEP** ]  on cultured rabbit growth plate chondrocytes.  Ref: 12876420 Horm Res, 2003
- Leptin  [ **LEPR** ]   [ **LEP** ]  accelerates gonadotropin releasing hormone GnRH pulsatility in hypothalamic neurons, and it has a direct effect on the anterior pituitary.  Ref: 12861226 Int J Obes Relat Metab Disord, 2003
- It has been shown that serum leptin  [ **LEPR** ]   [ **LEP** ]  levels correlate with weight and percentage body fat in normal and obese individuals, but the exact correlation between leptin  [ **LEPR** ]   [ **LEP** ]  and body weight in anorexic and bulimic patients has not yet been clarified.  Ref: 12880190 Eat Weight Disord, 2003
- Corticotropin releasing hormone mediated pathway of leptin  [ **LEPR** ]   [ **LEP** ]  to regulate feeding, adiposity, and uncoupling protein expression in mice.  Ref: 12865337 Endocrinology, 2003
- In peripheral tissue, leptin  [ **LEPR** ]   [ **LEP** ]  up regulated BAT UCP1 mRNA expression and reduced fat depositions in this tissue.  Ref: 12865337 Endocrinology, 2003
- Leptin  [ **LEPR** ]   [ **LEP** ]  a potential marker of placental insufficiency.  Ref: 12865594 Gynecol Obstet Invest, 2003
- The aim of the present study was to investigate the role of transcription and translation in the regulation of basal and insulin stimulated leptin  [ **LEPR** ]   [ **LEP** ]  production.  Ref: 12878197 Biochem Biophys Res CommunBiochem Biophys Res Commun, 2003
- Impact of dietary FA and energy restriction on plasma leptin  [ **LEPR** ]   [ **LEP** ]  and ob gene expression in mice.  Ref: 12880106 Lipids, 2003
- Leptin  [ **LEPR** ]   [ **LEP** ]  is a regulator of body weight and affects nitric oxide NO production.  Ref: 12869380 Am J Physiol Heart Circ PhysiolAm J Physiol Heart Circ Physiol, 2003
- We studied serum leptin  [ **LEPR** ]   [ **LEP** ] , TSH, fT4 and fT3 levels in 38 obese patients 26 women and 12 men, before and 12 months after BPD.  Ref: 12880185 Eat Weight Disord, 2003
- RESULTS There were no significant differences in serum E 2, P4 and leptin  [ **LEPR** ]   [ **LEP** ]  levels and follicular leptin  [ **LEPR** ]   [ **LEP** ]  and IGF I levels.  Ref: 12860336 Eur J Obstet Gynecol Reprod Biol, 2003
- OBJECTIVE This study aimed to evaluate whether leptin  [ **LEPR** ]   [ **LEP** ]  has any effect on growth plate chondrocytes.  Ref: 12876420 Horm Res, 2003
- In addition, TCF 4N and beta catenin synergized with the adipogenic transcription factor CCAAT enhancer binding protein alpha C EBPalpha to induce leptin  [ **LEPR** ]   [ **LEP** ]  promoter activity.  Ref: 12861022 Mol Cell Biol, 2003
- Infusion of insulin 1 micro g kg body weight per hour caused a progressive rise in the plasma concentration of leptin  [ **LEPR** ]   [ **LEP** ]  that reached maximum levels at 24 h during both physiological states.  Ref: 12881203 Am J Physiol Regul Integr Comp PhysiolAm J Physiol Regul Integr Comp Physiol, 2003
- We previously showed that this energy deficit contributes to a decline in plasma leptin  [ **LEPR** ]   [ **LEP** ] .  Ref: 12881203 Am J Physiol Regul Integr Comp PhysiolAm J Physiol Regul Integr Comp Physiol, 2003
- Role of obesity and leptin  [ **LEPR** ]   [ **LEP** ]  in the pubertal process and pubertal growth a review.  Ref: 12861226 Int J Obes Relat Metab Disord, 2003
- In patients with acromegaly, total leptin  [ **LEPR** ]   [ **LEP** ]  increases following successful pituitary surgery and during somatostatin SMS analogue therapy despite no change in BMI but whether this represents changes in soluble leptin  [ **LEPR** ]   [ **LEP** ]  receptor, bound or free leptin  [ **LEPR** ]   [ **LEP** ]  is unclear.  Ref: 12864793 Clin Endocrinol (Oxf)Clin Endocrinol (Oxf), 2003
- Alterations in Nitric Oxide cyclic GMP Pathway in Ventricular Myocytes from Obese Leptin  [ **LEPR** ]   [ **LEP** ]  deficient Mice.  Ref: 12869380 Am J Physiol Heart Circ PhysiolAm J Physiol Heart Circ Physiol, 2003
- The response of the hypothalamic pituitary gonadal axis to fasting is modulated by leptin  [ **LEPR** ]   [ **LEP** ] .  Ref: 12856798 Endocr Res, 2003
- ... when ewes were fed to appetite throughout gestation, fetal adipose tissue deposition and leptin  [ **LEPR** ]   [ **LEP** ]  mRNA abundance were both reduced.  Ref: 12865340 Endocrinology, 2003
- These results suggest that leptin  [ **LEPR** ]   [ **LEP** ]  is not only an adipostatic hormone but also a stress related hormone.  Ref: 12880122 J Pediatr Endocrinol Metab, 2003
- The increase of beta LH mRNA seen in the fasting state was prevented by leptin  [ **LEPR** ]   [ **LEP** ] .  Ref: 12856798 Endocr Res, 2003
- Central infusion of leptin  [ **LEPR** ]   [ **LEP** ]  into the lateral cerebroventricle icv caused significant induction of c FLI in the paraventricular nucleus PVN, ventromedial hypothalamic nucleus VMH, dorsomedial hypothalamic nucleus, and arcuate nucleus.  Ref: 12865337 Endocrinology, 2003
- Expression levels for these genes, except for leptin  [ **LEPR** ]   [ **LEP** ]  receptor, showed less in chorionic villi from RPL patients than those from normal controls.  Ref: 12874795 Mol Reprod Dev, 2003
- Two groups of male rats, aged 65 days old, were either fasted and saline injected or fasted and leptin  [ **LEPR** ]   [ **LEP** ]  treated for approximately three days.  Ref: 12856798 Endocr Res, 2003
- After normalization of serum IGF I, a correlation between BMI and leptin  [ **LEPR** ]   [ **LEP** ]  remained R = 0.86, P &lt.  Ref: 12864793 Clin Endocrinol (Oxf)Clin Endocrinol (Oxf), 2003
- Some recent discoveries have made it evident that this is a very active endocrine tissue that secretes important molecules related to different processes such as the immune response TNFa the regulation of food intake and expenditure of energy leptin  [ **LEPR** ]   [ **LEP** ] , Acrp30 adipoQ and the vascular function angiotensin and plasminogen activator inhibitor type 1.  Ref: 12861268 An Sist Sanit NavarAn Sist Sanit Navar, 2003
- When LH microdialysates were collected in the absence of food intake, neither artificial cerebrospinal fluid CSF nor 10 μg leptin  [ **LEPR** ]   [ **LEP** ]  i.c.v. caused significant variations in 5 HT release, measured for 2 h post injection, at 20 min periods.  Ref: 12873793 Regul PeptRegul Pept, 2003
- Leptin  [ **LEPR** ]   [ **LEP** ]  was able to prevent the fasting induced fall of serum testosterone.  Ref: 12856798 Endocr Res, 2003
- Serum levels of TC, TG, LDL C and insulin were significantly higher in obese leptin  [ **LEPR** ]   [ **LEP** ]  resistant group than those in normally weighted control group, but no significant difference in them between obese leptin  [ **LEPR** ]   [ **LEP** ]  sensitive group and its normally weighted control group was observed.  Ref: 12880566 Zhonghua Yu Fang Yi Xue Za Zhi, 2003
- Both hypothalamic serotonin and leptin  [ **LEPR** ]   [ **LEP** ]  reduce energy intake and stimulate expenditure.  Ref: 12873793 Regul PeptRegul Pept, 2003
- Maternal Endocrine Adaptation throughout Pregnancy to Nutritional Manipulation Consequences for Maternal Plasma Leptin  [ **LEPR** ]   [ **LEP** ]  and Cortisol and the Programming of Fetal Adipose Tissue Development.  Ref: 12865340 Endocrinology, 2003
- The correlations between plasma leptin  [ **LEPR** ]   [ **LEP** ]  levels and left ventricular mass index, sum of wall thicknesses, and blood pressure were calculated.  Ref: 12872039 J HypertensJ Hypertens, 2003
- E 2, P4, leptin  [ **LEPR** ]   [ **LEP** ]  and IGF I levels in serum or follicular fluid FF were measured with radioimmunoassay.  Ref: 12860336 Eur J Obstet Gynecol Reprod Biol, 2003
- Leptin  [ **LEPR** ]   [ **LEP** ]  resistance correlated significantly with the risk of metabolic syndrome and cardiovascular disease, indicating serum level of leptin  [ **LEPR** ]   [ **LEP** ]  could be used as an indicator in screening obese children at high risk.  Ref: 12880566 Zhonghua Yu Fang Yi Xue Za Zhi, 2003
- 0.01, improvement in glucose tolerance without a change in serum insulin levels, and an increase in serum leptin  [ **LEPR** ]   [ **LEP** ]  levels in comparison with control 2.6 0.3 ng ml vs. 1.7 0.2 ng ml, p &lt.  Ref: 12878760 J PhysiolJ Physiol, 2003
- Although bulimic patients have very bad nutritional behaviour, their leptin  [ **LEPR** ]   [ **LEP** ]  levels do not appear altered.  Ref: 12880190 Eat Weight Disord, 2003
- These results indicated that the NO cGMP signaling pathway was significantly altered in ventricular myocytes from the leptin  [ **LEPR** ]   [ **LEP** ]  deficient obese mouse model.  Ref: 12869380 Am J Physiol Heart Circ PhysiolAm J Physiol Heart Circ Physiol, 2003
- Leptin  [ **LEPR** ]   [ **LEP** ]  was also inversely related to the sum of wall thicknesses.  Ref: 12872039 J HypertensJ Hypertens, 2003
- The aim of the study was to evaluate the possible correlations between leptin  [ **LEPR** ]   [ **LEP** ]  levels and the body mass index BMI in all five groups.  Ref: 12880190 Eat Weight Disord, 2003
- Plasma leptin  [ **LEPR** ]   [ **LEP** ]  levels in patients with burn injury a preliminary report.  Ref: 12880724 Burns, 2003
- 0.0001 and together BMI and gender accounted for 87% of the variance in mean log10 serum leptin  [ **LEPR** ]   [ **LEP** ]  P = 0.0002.  Ref: 12864793 Clin Endocrinol (Oxf)Clin Endocrinol (Oxf), 2003
- Similar to testosterones dependence on leptin  [ **LEPR** ]   [ **LEP** ] , leptin  [ **LEPR** ]   [ **LEP** ]  concentrations were somewhat dependent on testosterone.  Ref: 12856798 Endocr Res, 2003
- Actinomycin D 5μg ml increased both basal and insulin stimulated 1.6nM leptin  [ **LEPR** ]   [ **LEP** ]  secretion at 4 and 24h 193 14.9% and 153.8 10.4% of respective controls at 24h, both p< ; 0.001.  Ref: 12878197 Biochem Biophys Res CommunBiochem Biophys Res Commun, 2003
- A blood sample was taken for the analysis of free and total thyroxine, TSH, and leptin  [ **LEPR** ]   [ **LEP** ] .  Ref: 12857676 Am J Physiol Endocrinol Metab, 2003
- and for leptin  [ **LEPR** ]   [ **LEP** ]  on chromosome 3.  Ref: 12865502 Physiol GenomicsPhysiol Genomics, 2003
- The dose response experiment showed that the maximal effect of leptin  [ **LEPR** ]   [ **LEP** ]  on insulin secretion achieved at 2 nmol L.  Ref: 12877797 Chin Med J (Engl), 2003
- We utilized a mouse model to examine the effects of intermittent hypoxia on insulin resistance in lean C57BL 6J mice and leptin  [ **LEPR** ]   [ **LEP** ]  deficient obese C57BL 6J  **Lep**  ob mice.  Ref: 12878760 J PhysiolJ Physiol, 2003
- RESULTS As in normal subjects, females with acromegaly had higher baseline serum leptin  [ **LEPR** ]   [ **LEP** ]  median M = 6.1 range 1.6 58.7, F = 25.9 range 3.19 54.1 ng ml.  Ref: 12864793 Clin Endocrinol (Oxf)Clin Endocrinol (Oxf), 2003
- There are numerous future perspectives amongst which are leptin  [ **LEPR** ]   [ **LEP** ] , analogs of GLP 1 and cholecystokinin and neuropeptide Y antagonists.  Ref: 12861278 An Sist Sanit Navar, 2002
- Low circulating leptin  [ **LEPR** ]   [ **LEP** ]  in response to weight loss in any gastrointestinal disease may be an important factor in reducing bone mass.  Ref: 12867794 Eur J Gastroenterol Hepatol, 2003
- In addition, there was a strong, negative, linear relation between whole body insulin sensitivity based on postprandial responses of glucose and insulin and postprandial leptin  [ **LEPR** ]   [ **LEP** ]  concentrations at 6 h.  Ref: 12861230 Int J Obes Relat Metab DisordInt J Obes Relat Metab Disord, 2003
- RESULTS In the combined treatment group, the intake volume of water and food were reduced, leading to weight loss and undetectable levels of leptin  [ **LEPR** ]   [ **LEP** ]  in the serum.  Ref: 12875675 Chin Med J (Engl), 2003
- Protein tyrosine phosphatase PTPase 1B PTP1B has been implicated as a key negative regulator of both insulin and leptin  [ **LEPR** ]   [ **LEP** ]  signaling cascades.  Ref: 12877578 J Med Chem, 2003
- p< ; 0.01 and insulin stimulated leptin  [ **LEPR** ]   [ **LEP** ]  secretion 141.0 11.1% of insulin treated cells.  Ref: 12878197 Biochem Biophys Res CommunBiochem Biophys Res Commun, 2003
- Besides, hormones such as insulin and leptin  [ **LEPR** ]   [ **LEP** ]  act over the longer term on the brain, reducing ingestion and increasing energy expenditure.  Ref: 12861269 An Sist Sanit Navar, 2002
- Circulating leptin  [ **LEPR** ]   [ **LEP** ]  levels were also substantially lower in lean 45% less and nonlean 63% less, both P< ; 0.0001 ultramarathon runners.  Ref: 12869354 Arterioscler Thromb Vasc BiolArterioscler Thromb Vasc Biol, 2002
- The principal aim of this study was to evaluate serum leptin  [ **LEPR** ]   [ **LEP** ]  concentrations and to analyze the interaction between serum leptin  [ **LEPR** ]   [ **LEP** ]  levels and C reactive protein CRP levels, hematological parameters before and after antimicrobial therapy in neonates with bacterial septicemia.  Ref: 12880122 J Pediatr Endocrinol Metab, 2003
- 31% fat diet for 10d, DIO rats ate 70% more, gained more body and adipose depot weight, had higher leptin  [ **LEPR** ]   [ **LEP** ]  and insulin levels and had 22% lower feed efficiency than DR rats fed HE diet.  Ref: 12865257 Am J Physiol Endocrinol MetabAm J Physiol Endocrinol Metab, 2003
- CRP and leptin  [ **LEPR** ]   [ **LEP** ]  were measured by ELISA and radioimmunoassay, respectively.  Ref: 12869354 Arterioscler Thromb Vasc BiolArterioscler Thromb Vasc Biol, 2003
- Serum levels of leptin  [ **LEPR** ]   [ **LEP** ]  were detected with an enzyme linked immuno sorbent assay ELISA.  Ref: 12875675 Chin Med J (Engl), 2003
- Similar effects of actinomycin D were observed on basal and insulin stimulated leptin  [ **LEPR** ]   [ **LEP** ]  mRNA levels.  Ref: 12878197 Biochem Biophys Res CommunBiochem Biophys Res Commun, 2003
- BACKGROUND AND OBJECTIVE Leptin  [ **LEPR** ]   [ **LEP** ]  is produced by the human placenta besides adipose tissue and is suggested to be related to fetal growth.  Ref: 12880123 J Pediatr Endocrinol Metab, 2003
- Thus, short term administration of a diet rich in SAFA suppresses circulating leptin  [ **LEPR** ]   [ **LEP** ]  levels without altering the adipose tissue ob gene expression.  Ref: 12880106 Lipids, 2003
- These data support the hypothesis that leptin  [ **LEPR** ]   [ **LEP** ]  plays a specific role in mediating the response of reproductive hormones to the nutritional status of the organism.  Ref: 12856798 Endocr Res, 2003
- Elevated leptin  [ **LEPR** ]   [ **LEP** ]  levels might have a permissive effect on the pubertal process and pubertal growth.International Journal of Obesity 2003 2 69 874.  Ref: 12861226 Int J Obes Relat Metab Disord, 2003
- Long life span was predicted by low levels of leptin  [ **LEPR** ]   [ **LEP** ]  at age 4 months in females, and by low levels of IGF I at age 15 months and high levels of T 4 at age 4 months, in males.  Ref: 12875743 Mech Ageing Dev, 2003
- We believe that, although thyroid hormones and lipid metabolic products do not seem to participate in the regulation of serum leptin  [ **LEPR** ]   [ **LEP** ]  levels, BMI and insulin resistance status may have a key role in women with PCOS.  Ref: 12857430 Gynecol Endocrinol, 2003
- Fasting serum levels of thyroid stimulating hormone TSH, free triiodothyronine FT3, free thyroxine FT4, fasting glucose, insulin, total cholesterol TC, triglyceride TG, high density lipoprotein cholesterol HDL C, low density lipoprotein cholesterol LDL C, very low density lipoprotein cholesterol VLDL C and leptin  [ **LEPR** ]   [ **LEP** ]  were measured and compared in the three groups and the correlations between serum levels of leptin  [ **LEPR** ]   [ **LEP** ]  and other parameters were evaluated.  Ref: 12857430 Gynecol Endocrinol, 2003
- 0.001 and a 5 fold increase in serum leptin  [ **LEPR** ]   [ **LEP** ]  levels p&lt.  Ref: 12869545 J Biol ChemJ Biol Chem, 2003
- During lactation, mammary epithelial cells expressed leptin  [ **LEPR** ]   [ **LEP** ]  mRNA, but insulin did not increase milk leptin  [ **LEPR** ]   [ **LEP** ]  output.  Ref: 12881203 Am J Physiol Regul Integr Comp PhysiolAm J Physiol Regul Integr Comp Physiol, 2003
- CONCLUSION Daily moderate intensity exercise, without concomitant changes in body fat mass, suppressed fasting and postprandial circulating leptin  [ **LEPR** ]   [ **LEP** ]  concentrations after consumption of a short term high carbohydrate diet.  Ref: 12861230 Int J Obes Relat Metab DisordInt J Obes Relat Metab Disord, 2003
- The regulation could be direct through an action of glucocorticoid receptors or indirectly through ADX induced changes in leptin  [ **LEPR** ]   [ **LEP** ]  levels.  Ref: 12872289 Synapse, 2003
- A significant correlation was observed between serum leptin  [ **LEPR** ]   [ **LEP** ]  levels and both BMI and insulin resistance status in PCOS.  Ref: 12857430 Gynecol Endocrinol, 2003
- However, leptin  [ **LEPR** ]   [ **LEP** ]  levels remained within the normal range, except in a patient Case 1 complicated with severe hypovolemic shock.  Ref: 12880724 Burns, 2003
- ARA improved insulin resistance, and reduced plasma leptin  [ **LEPR** ]   [ **LEP** ]  and leptin  [ **LEPR** ]   [ **LEP** ]  mRNA in adipose tissue.  Ref: 12862206 Hypertens Res, 2003
- Plasmalemmal fatty acid transport is regulated in heart and skeletal muscle by contraction, insulin and leptin  [ **LEPR** ]   [ **LEP** ] , and in obesity and diabetes.  Ref: 12864739 Acta Physiol Scand, 2003
- Homeostasis model assessment insulin resistance HOMA IR index, plasma levels of leptin  [ **LEPR** ]   [ **LEP** ] , and leptin  [ **LEPR** ]   [ **LEP** ]  mRNA in mesenteric adipose tissue were significantly greater in the sucrose rich chow fed animals than in the standard chow fed animals, and significantly lower in the ARA treated sucrose rich chow fed animals than in the sucrose rich chow fed animals in both SHR and WKY.  Ref: 12862206 Hypertens Res, 2003
- The effect of pegvisomant induced serum IGF I normalization on serum leptin  [ **LEPR** ]   [ **LEP** ]  levels in patients with acromegaly.  Ref: 12864793 Clin Endocrinol (Oxf)Clin Endocrinol (Oxf), 2003
- Castration accelerated the normal, age related increase in serum leptin  [ **LEPR** ]   [ **LEP** ] .  Ref: 12856798 Endocr Res, 2003
- Seasonal variation in sleeping metabolic rate, thyroid activity, and leptin  [ **LEPR** ]   [ **LEP** ] .  Ref: 12857676 Am J Physiol Endocrinol Metab, 2003
- Effects of angiotensin II receptor antagonists on insulin resistance syndrome and leptin  [ **LEPR** ]   [ **LEP** ]  in sucrose fed spontaneously hypertensive rats.  Ref: 12862206 Hypertens Res, 2003
- Quantitative Trait Loci for Insulin like Growth Factor I, Leptin  [ **LEPR** ]   [ **LEP** ] , Thyroxine, and Corticosterone in Genetically Heterogeneous Mice.  Ref: 12865502 Physiol GenomicsPhysiol Genomics, 2003
- At the 0 20 min interval, serotonin release was significantly higher after leptin  [ **LEPR** ]   [ **LEP** ]  42 12% above baseline than after CSF 6 5% and the maximal increase after leptin  [ **LEPR** ]   [ **LEP** ]  was of 126 53% above baseline 100 120 min, p> ; 0.05 vs. CSF.  Ref: 12873793 Regul PeptRegul Pept, 2003
- The mean concentration of serum leptin  [ **LEPR** ]   [ **LEP** ] , CRP levels, and immature total neutrophil IT ratio in newborns with septicemia were significantly higher than those of controls at the start.  Ref: 12880122 J Pediatr Endocrinol Metab, 2003
- This indicates that saturated fat may alter protein handling by adipose tissue or the whole body clearance of leptin  [ **LEPR** ]   [ **LEP** ] .  Ref: 12880106 Lipids, 2003
- Effects of inhibiting transcription and protein synthesis on basal and insulin stimulated leptin  [ **LEPR** ]   [ **LEP** ]  gene expression and leptin  [ **LEPR** ]   [ **LEP** ]  secretion in cultured rat adipocytes.  Ref: 12878197 Biochem Biophys Res CommunBiochem Biophys Res Commun, 2003
- The CLA isomers and fenofibrate altered mRNA levels for several proteins involved in lipid metabolism, but the most striking difference was the reduction of mRNA for leptin  [ **LEPR** ]   [ **LEP** ]  and adiponectin in the t10, c12 CLA group.  Ref: 12880104 Lipids, 2003
- Protein tyrosine phosphatase 1B PTP1B has been implicated as one of the key negative regulators of insulin and leptin  [ **LEPR** ]   [ **LEP** ]  signal transduction pathways.  Ref: 12871138 Curr Med Chem, 2003
- Here, chow fed DIO rats ate 9% more and gained 19% more body weight from 5 6wk of age than did DR rats but had comparable leptin  [ **LEPR** ]   [ **LEP** ]  and insulin levels.  Ref: 12865257 Am J Physiol Endocrinol MetabAm J Physiol Endocrinol Metab, 2003
- Serum samples were taken at 24 hours following delivery and leptin  [ **LEPR** ]   [ **LEP** ]  concentrations were measured.  Ref: 12880123 J Pediatr Endocrinol Metab, 2003
- These observations indicate that leptin  [ **LEPR** ]   [ **LEP** ]  probably interacts with the serotonergic stimulating mechanisms elicited by food intake, intensifying them.  Ref: 12873793 Regul PeptRegul Pept, 2003
- Plasma levels were monitored for leptin  [ **LEPR** ]   [ **LEP** ] , proinflammatory cytokines interleukin IL 1beta, IL 6, tumor necrosis factor alpha TNF alpha, stress related parameters adrenocorticotropic hormone ACTH, cortisol, and C reactive protein CRP.  Ref: 12880724 Burns, 2003
- Leptin  [ **LEPR** ]   [ **LEP** ]  also prevented the fasting induced fall in luteinizing hormone LH.  Ref: 12856798 Endocr Res, 2003
- Pegvisomant, a GH receptor antagonist capable of normalizing serum IGF I in over 97% of patients, represents a novel treatment strategy in acromegaly and its effect on leptin  [ **LEPR** ]   [ **LEP** ]  has not previously been reported.  Ref: 12864793 Clin Endocrinol (Oxf)Clin Endocrinol (Oxf), 2003
- Leptin  [ **LEPR** ]   [ **LEP** ]  inhibited food intake 75% at 0 20 min and 73% at 20 40 min while it accentuated the food induced serotonergic activation.  Ref: 12873793 Regul PeptRegul Pept, 2003
- The additional serotonergic activation induced by leptin  [ **LEPR** ]   [ **LEP** ]  may be significant for the hormone effects on energy balance.  Ref: 12873793 Regul PeptRegul Pept, 2003
- Leptin  [ **LEPR** ]   [ **LEP** ]  is an adipocyte derived signal factor 167 amino acid protein encoded by the ob gene in chromosome 7q31 that regulates eating behaviour via central neuroendocrine mechanisms.  Ref: 12880190 Eat Weight Disord, 2003
- In all these nuclei, the effect of leptin  [ **LEPR** ]   [ **LEP** ]  on expression of cFLI in the PVN and VMH was decreased by treatment with alphaCRH.  Ref: 12865337 Endocrinology, 2003
- CONCLUSION Pegvisomant induced serum IGF I normalization in patients with active acromegaly is associated with a significant increase in total, and by implication, free leptin  [ **LEPR** ]   [ **LEP** ] .  Ref: 12864793 Clin Endocrinol (Oxf)Clin Endocrinol (Oxf), 2003
- The purified islets were incubated with different concentrations of leptin  [ **LEPR** ]   [ **LEP** ]  for 2 h in the presence of different concentrations of glucose.  Ref: 12877797 Chin Med J (Engl), 2003
- Forward step wise regression analysis demonstrated that BMI and gender accounted for 90% of the variance in mean serum log10 leptin  [ **LEPR** ]   [ **LEP** ] .  Ref: 12864793 Clin Endocrinol (Oxf)Clin Endocrinol (Oxf), 2003
- Abnormalities of Leptin  [ **LEPR** ]   [ **LEP** ]  and Ghrelin Regulation in Obesity prone Juvenile Rats.  Ref: 12865257 Am J Physiol Endocrinol MetabAm J Physiol Endocrinol Metab, 2003
- Placental leptin  [ **LEPR** ]   [ **LEP** ]  content was increased 3 fold in group 1 compared to group 2 192.5.1 39.5 vs. 67.8 10.6 ng g and 8 fold in group 1 compared to group 3 192.5.1 39.5 vs. 25.4 6.9 ng g.  Ref: 12865594 Gynecol Obstet Invest, 2003
- In contrast, a three day period of GH administration had no effect on plasma leptin  [ **LEPR** ]   [ **LEP** ]  during LP or EL.  Ref: 12881203 Am J Physiol Regul Integr Comp PhysiolAm J Physiol Regul Integr Comp Physiol, 2003
- Our group has shown that leptin  [ **LEPR** ]   [ **LEP** ]  acts as a skeletal growth factor, with a direct effect on skeletal growth centers, in the mice mandibular condyle, a model of endochondral ossification.  Ref: 12861226 Int J Obes Relat Metab Disord, 2003
- Leptin  [ **LEPR** ]   [ **LEP** ]  receptors have been identified in the hypothalamus, gonadotrope cells of the anterior pituitary, and ovarian follicular cells, as well as Leydig cells.  Ref: 12861226 Int J Obes Relat Metab Disord, 2003
- Overall fasted and postprandial states, plasma leptin  [ **LEPR** ]   [ **LEP** ]  concentrations were significantly higher after the High CHO than after the High CHO Ex intervention.  Ref: 12861230 Int J Obes Relat Metab DisordInt J Obes Relat Metab Disord, 2003
- Chronic leptin  [ **LEPR** ]   [ **LEP** ]  treatment decreases LCFA transporters and transport in muscle.  Ref: 12864739 Acta Physiol Scand, 2003
- A possible role of thyroid hormones in regulating adipose tissue metabolism in humans has been proposed, but it is not universally accepted and the relationship between thyroid function and leptin  [ **LEPR** ]   [ **LEP** ]  levels has not yet been clearly defined.  Ref: 12880185 Eat Weight Disord, 2003
- However, serum leptin  [ **LEPR** ]   [ **LEP** ]  concentrations of twins mean SEM 1.13 0.35 ng ml were significantly lower than of the singleton infants 4.27 0.63 ng ml p <.  Ref: 12880123 J Pediatr Endocrinol Metab, 2003
- Administration of leptin  [ **LEPR** ]   [ **LEP** ]  markedly decreased cumulative food intake and body weight with this effect being attenuated by pretreatment with alphaCRH.  Ref: 12865337 Endocrinology, 2003
- Leptin  [ **LEPR** ]   [ **LEP** ]  was not statistically associated with blood pressure after adjusting for body mass index.CONCLUSIONS The results do not support the hypothesis that leptin  [ **LEPR** ]   [ **LEP** ]  increases cardiovascular risk by increasing left ventricular mass index or blood pressure.  Ref: 12872039 J HypertensJ Hypertens, 2003
- The role of leptin  [ **LEPR** ]   [ **LEP** ]  in the etiopathogenesis of anorexia nervosa and bulimia.  Ref: 12880190 Eat Weight Disord, 2003
- The effect of actinomycin D and DRB to increase basal leptin  [ **LEPR** ]   [ **LEP** ]  secretion observed at 4 and 24h was not present at 48h when actinomycin D and DRB both markedly inhibited insulin stimulated leptin  [ **LEPR** ]   [ **LEP** ]  secretion to 36 16%, p< ; 0.05 and 21.9 5.6% of control, for actinomycin D and DRB, respectively, both p< ; 0.001.  Ref: 12878197 Biochem Biophys Res CommunBiochem Biophys Res Commun, 2003
- Among candidate genes are genes encoding glycogen synthase, beta 3 adrenergic receptor, glycogen associated regulatory subunit of protein phosphatase 1, peroxisome proliferator activated receptor gamma PPAR gamma, leptin  [ **LEPR** ]   [ **LEP** ]  and adiponectin.  Ref: 12877070 Nippon Rinsho, 2003
- The plasma leptin  [ **LEPR** ]   [ **LEP** ]  concentrations on ad libitum CH and MUFA diets did not differ significantly 24.3 2.1 and 34.7 6.7 ng mL, respectively whereas the saturated fat diet caused a lower concentration 13.9 1.9 ng mL.  Ref: 12880106 Lipids, 2003
- OBJECTIVE This study was undertaken to evaluate to what extent C reactive protein CRP can be reduced by exercise by examining its circulating concentrations in male ultramarathon runners and to determine if low leptin  [ **LEPR** ]   [ **LEP** ]  as a robust circulating marker of fat mass could account for low CRP in such men.  Ref: 12869354 Arterioscler Thromb Vasc BiolArterioscler Thromb Vasc Biol, 2003
- This decline mirrors that of plasma insulin, but is reciprocal to the profile of plasma growth hormone GH, suggesting that both hormones may regulate plasma leptin  [ **LEPR** ]   [ **LEP** ]  in periparturient dairy cows.  Ref: 12881203 Am J Physiol Regul Integr Comp PhysiolAm J Physiol Regul Integr Comp Physiol, 2003
- OBJECTIVE To investigate the relationship between fasting serum levels of leptin  [ **LEPR** ]   [ **LEP** ] , glucose, insulin resistance, lipids in simple obese children.  Ref: 12880566 Zhonghua Yu Fang Yi Xue Za Zhi, 2003
- Microarray mRNA analysis in adipose tissue revealed that leptin  [ **LEPR** ]   [ **LEP** ]  was the only up regulated gene affecting glucose uptake.  Ref: 12878760 J PhysiolJ Physiol, 2003
- Multiple linear regression analysis was used to adjust for other cardiovascular risk factors.RESULTS After adjusting for age, body mass index, systolic blood pressure, sex, and insulin resistance, leptin  [ **LEPR** ]   [ **LEP** ]  was inversely associated with left ventricular mass index beta = 0.20, P &lt.  Ref: 12872039 J HypertensJ Hypertens, 2003
- Our aim was to determine whether serum leptin  [ **LEPR** ]   [ **LEP** ]  level is regulated by thyroid hormones, lipid metabolic products and insulin resistance status in women with polycystic ovary syndrome PCOS.  Ref: 12857430 Gynecol Endocrinol, 2003
- 0.001 and leptin  [ **LEPR** ]   [ **LEP** ]  P <.  Ref: 12857676 Am J Physiol Endocrinol Metab, 2003
- Is serum leptin  [ **LEPR** ]   [ **LEP** ]  level regulated by thyroid functions, lipid metabolism and insulin resistance in polycystic ovary syndrome ? .  Ref: 12857430 Gynecol Endocrinol, 2003
- Insulin infusion increased leptin  [ **LEPR** ]   [ **LEP** ]  mRNA in adipose tissue during LP but not during EL.  Ref: 12881203 Am J Physiol Regul Integr Comp PhysiolAm J Physiol Regul Integr Comp Physiol, 2003
- This study was designed to determine whether the myocardial NO cGMP signal transduction system was altered in leptin  [ **LEPR** ]   [ **LEP** ]  deficient obese mice.  Ref: 12869380 Am J Physiol Heart Circ PhysiolAm J Physiol Heart Circ Physiol, 2003
- We investigated leptin  [ **LEPR** ]   [ **LEP** ]  levels in the serum of 58 female subjects aged 15 36 years 10 with bulimia nervosa BN.  Ref: 12880190 Eat Weight Disord, 2003
- The stimulatory regulation of SUR1 transcription levels may be mediated through activation of PI 3 kinase pathway, which may be a possible mechanism of leptin  [ **LEPR** ]   [ **LEP** ]  in regulating insulin secretion.  Ref: 12877797 Chin Med J (Engl), 2003
- Other factors, such as increased response of adipose tissue to beta adrenergic signals, probably contribute to the reduction of plasma leptin  [ **LEPR** ]   [ **LEP** ]  in early lactating dairy cows.  Ref: 12881203 Am J Physiol Regul Integr Comp PhysiolAm J Physiol Regul Integr Comp Physiol, 2003
- Recent data indicate that leptin  [ **LEPR** ]   [ **LEP** ]  has a specific role in stimulating the activity of enzymes essential for the synthesis of adrenal androgens.  Ref: 12861226 Int J Obes Relat Metab Disord, 2003
- Leptin  [ **LEPR** ]   [ **LEP** ]  administration at low doses may have a permissive, threshold effect on the central networks that regulate gonadotropin secretion.  Ref: 12861226 Int J Obes Relat Metab Disord, 2003
- These results suggest that the plasma leptin  [ **LEPR** ]   [ **LEP** ]  level may have some relations to plasma proimflammatory cytokines in pathophysiologic responses to critical conditions of burn injury.  Ref: 12880724 Burns, 2003
- Of obese children, 83% were leptin  [ **LEPR** ]   [ **LEP** ]  resistant.  Ref: 12880566 Zhonghua Yu Fang Yi Xue Za Zhi, 2003
- Serum leptin  [ **LEPR** ]   [ **LEP** ]  levels in neonatal bacterial septicemia.  Ref: 12880122 J Pediatr Endocrinol Metab, 2003
- Serum leptin  [ **LEPR** ]   [ **LEP** ]  levels were higher in the O PCOS group, while its level was comparable between the L PCOS and control groups.  Ref: 12857430 Gynecol Endocrinol, 2003
- Relationship between serum levels of leptin  [ **LEPR** ]   [ **LEP** ]  and glucose, lipids in simple obese children .  Ref: 12880566 Zhonghua Yu Fang Yi Xue Za Zhi, 2003
- Although there were positive correlations between serum leptin  [ **LEPR** ]   [ **LEP** ]  levels and serum CRP levels and IT ratio in the septicemic group at the start, there were no correlations between serum leptin  [ **LEPR** ]   [ **LEP** ]  levels and other hematological parameters.  Ref: 12880122 J Pediatr Endocrinol Metab, 2003
- The short term 4h and long term 24 48h effects of actinomycin D, a transcriptional inhibitor, and cycloheximide, an inhibitor of protein synthesis, on leptin  [ **LEPR** ]   [ **LEP** ]  gene expression and leptin  [ **LEPR** ]   [ **LEP** ]  secretion by isolated adipocytes were determined.  Ref: 12878197 Biochem Biophys Res CommunBiochem Biophys Res Commun, 2003
- Serum levels of thyroxine T 4, leptin  [ **LEPR** ]   [ **LEP** ] , and insulin like growth factor I IGF I, as well as cataract severity, were evaluated as predictors of life span in a population of genetically heterogeneous mice UM HET3.  Ref: 12875743 Mech Ageing Dev, 2003
- Pegvisomant induced serum IGF I normalization was associated with a rise in serum leptin  [ **LEPR** ]   [ **LEP** ]  8.9 range 1.6 58.3 to 12.7 range 2.3 90.8 ng ml, P &lt.  Ref: 12864793 Clin Endocrinol (Oxf)Clin Endocrinol (Oxf), 2003
- Cycloheximide treatment 10μg ml abolished the effects of insulin to stimulate leptin  [ **LEPR** ]   [ **LEP** ]  secretion 29 11% of control, p< ; 0.01 during the first 4h of treatment and at all later time points, which indicate that de novo protein synthesis is required for insulin mediated glucose metabolism to increase leptin  [ **LEPR** ]   [ **LEP** ]  secretion.  Ref: 12878197 Biochem Biophys Res CommunBiochem Biophys Res Commun, 2003
- Effect of Insulin and Growth Hormone on Plasma Leptin  [ **LEPR** ]   [ **LEP** ]  in the Periparturient Dairy Cow.  Ref: 12881203 Am J Physiol Regul Integr Comp PhysiolAm J Physiol Regul Integr Comp Physiol, 2003
- Pregnancy resulted in a rise in plasma leptin  [ **LEPR** ]   [ **LEP** ]  concentration by 28 d gestation, which continued up to 80 d gestation when fed to appetite but not with nutrient restriction.  Ref: 12865340 Endocrinology, 2003
- Interestingly, no differences in plasma leptin  [ **LEPR** ]   [ **LEP** ]  levels between groups were seen in the energy restricted groups mean 8.0 1.0 ng mL.  Ref: 12880106 Lipids, 2003
- We conclude that the increase in insulin resistance in response to intermittent hypoxia is dependent on the disruption of leptin  [ **LEPR** ]   [ **LEP** ]  pathways.  Ref: 12878760 J PhysiolJ Physiol, 2003
- A single batch measurement of leptin  [ **LEPR** ]   [ **LEP** ] , bound leptin  [ **LEPR** ]   [ **LEP** ]  BL, soluble leptin  [ **LEPR** ]   [ **LEP** ]  receptor SLR, insulin and glucose were performed on samples from baseline and first occurrence of serum IGF I normalization.  Ref: 12864793 Clin Endocrinol (Oxf)Clin Endocrinol (Oxf), 2003
- Blood leptin  [ **LEPR** ]   [ **LEP** ]  levels were measured by radioimmune assay.  Ref: 12877823 Pulm Pharmacol Ther, 2003
- Furthermore, leptin  [ **LEPR** ]   [ **LEP** ]  adjustment only minimally attenuated the case control difference in CRP, suggesting that mechanisms other than fat mass reduction contribute to low concentrations of CRP in marathon runners.  Ref: 12869354 Arterioscler Thromb Vasc BiolArterioscler Thromb Vasc Biol, 2003
- METHODS Fasting serum levels of leptin  [ **LEPR** ]   [ **LEP** ]  and insulin Fins were measured by RIA in 42 obese and 42 normally weighted children matched on age, sex and height, and their total cholesterol TC, triglyceride TG, high density lipoprotein cholesterol HDL C were analyzed with enzymatic methods.  Ref: 12880566 Zhonghua Yu Fang Yi Xue Za Zhi, 2003
- Prior to semiconfluence, leptin  [ **LEPR** ]   [ **LEP** ]  increased 3 H thymidine incorporation while at the semiconfluent and early confluent stages, leptin  [ **LEPR** ]   [ **LEP** ]  promoted ALP activity and tended to promote proteoglycan production.  Ref: 12876420 Horm Res, 2003
- Taken together, these results indicate that CRH neurons in the PVN and VMH may be an important mediator for leptin  [ **LEPR** ]   [ **LEP** ]  that contribute to regulation of feeding, adiposity, and UCP expression.  Ref: 12865337 Endocrinology, 2003
- ... reduced leptin  [ **LEPR** ]   [ **LEP** ]  sensitivity might play a causal role.  Ref: 12865257 Am J Physiol Endocrinol MetabAm J Physiol Endocrinol Metab, 2003
- The association between leptin  [ **LEPR** ]   [ **LEP** ]  and left ventricular hypertrophy a population based cross sectional study.  Ref: 12872039 J HypertensJ Hypertens, 2003
- Using microdialysis, we directly assessed the effect of an intracerebroventricular leptin  [ **LEPR** ]   [ **LEP** ]  injection on 5 HT release in the lateral hypothalamus of normal rats.  Ref: 12873793 Regul PeptRegul Pept, 2003
- Exposure of islets to 2 nmol L leptin  [ **LEPR** ]   [ **LEP** ]  induced a significant increase of SUR1 transcription levels by 71% P <.  Ref: 12877797 Chin Med J (Engl), 2003
- MEASUREMENTS Fasting and postprandial plasma or serum concentrations of leptin  [ **LEPR** ]   [ **LEP** ] , glucose and insulin.  Ref: 12861230 Int J Obes Relat Metab DisordInt J Obes Relat Metab Disord, 2003
- There was a poor correlation between serum leptin  [ **LEPR** ]   [ **LEP** ] , and FT4, TC, TG, HDL C and VLDL C levels.  Ref: 12857430 Gynecol Endocrinol, 2003
- Therefore, insulin increases plasma leptin  [ **LEPR** ]   [ **LEP** ]  in LP by stimulating adipose tissue synthesis, but has only marginal effects in EL when cows are in negative energy balance.  Ref: 12881203 Am J Physiol Regul Integr Comp PhysiolAm J Physiol Regul Integr Comp Physiol, 2003
- CONCLUSION Twin pregnancy might be a factor affecting serum leptin  [ **LEPR** ]   [ **LEP** ]  concentrations in newborn infants.  Ref: 12880123 J Pediatr Endocrinol Metab, 2003
- In order to investigate the usefulness of angiotensin II type 1 receptor AT1 antagonists ARA in the treatment of hypertension with insulin resistance syndrome, we studied the effects of a high dose sucrose diet and ARA on insulin sensitivity, plasma lipids, and leptin  [ **LEPR** ]   [ **LEP** ]  in spontaneous hypertensive rats SHR and Wistar Kyoto rats WKY.  Ref: 12862206 Hypertens Res, 2003
- We investigated seasonal variation in sleeping metabolic rate SMR and the possible relation to body composition, thyroid activity, and leptin  [ **LEPR** ]   [ **LEP** ] .  Ref: 12857676 Am J Physiol Endocrinol Metab, 2003
- There was a strong, positive, linear relation between postprandial insulin responses and postprandial leptin  [ **LEPR** ]   [ **LEP** ]  concentrations at 6 h.  Ref: 12861230 Int J Obes Relat Metab DisordInt J Obes Relat Metab Disord, 2003
- This increase in insulin secretion after 5 days of intermittent hypoxia was completely abolished by prior leptin  [ **LEPR** ]   [ **LEP** ]  infusion.  Ref: 12878760 J PhysiolJ Physiol, 2003
- The aim of the present study was to elucidate whether the qualitative composition of dietary fat influences plasma leptin  [ **LEPR** ]   [ **LEP** ]  and adipose tissue ob gene expression differentially.  Ref: 12880106 Lipids, 2003
- Serum leptin  [ **LEPR** ]   [ **LEP** ]  levels in twins and singleton newborns.  Ref: 12880123 J Pediatr Endocrinol Metab, 2003
- The change in individual plasma leptin  [ **LEPR** ]   [ **LEP** ]  levels did not show similar pattern in all these patients.  Ref: 12880724 Burns, 2003
- it was the discovery of the leptin  [ **LEPR** ]   [ **LEP** ]  that opened the way for numerous investigations destined to lay bare the mechanisms involved in this homeostasis.  Ref: 12861270 An Sist Sanit NavarAn Sist Sanit Navar, 2003
- Other mechanisms, related to atherosclerosis, could explain the increased risk of cardiovascular diseases observed with high leptin  [ **LEPR** ]   [ **LEP** ]  levels.  Ref: 12872039 J HypertensJ Hypertens, 2003
- Our data suggest that, although significantly reduced, serum leptin  [ **LEPR** ]   [ **LEP** ]  levels in fasting anorexic patients are non linearly related to body weight BMI.  Ref: 12880190 Eat Weight Disord, 2003
- Serum leptin  [ **LEPR** ]   [ **LEP** ]  was significantly p< ; 0.001 reduced in the anorexic patients because of the dramatic decrease in adipose mass caused by the nutritional defect, as is further supported by the significantly p< ; 0.001 low level of transferrinemia.  Ref: 12880190 Eat Weight Disord, 2003
- Two weeks after treatment, serum leptin  [ **LEPR** ]   [ **LEP** ]  levels in newborns with septicemia had decreased and were similar to those of controls.  Ref: 12880122 J Pediatr Endocrinol Metab, 2003
- Evaluation of serum leptin  [ **LEPR** ]   [ **LEP** ]  levels and thyroid function in morbidly obese patients treated with bariatric surgery.  Ref: 12880185 Eat Weight Disord, 2003
- Angiogenesis related genes that showed aberrant expression level are matrix metalloproteinase 2 MMP 2, plasminogen activator inhibitor PAI, integrin, transforming growth factor beta TGF beta, vascular endothelial growth factor VEGF, basic fibroblast growth factor bFGF, and leptin  [ **LEPR** ]   [ **LEP** ]  receptor.  Ref: 12874795 Mol Reprod Dev, 2003
- Effect of leptin  [ **LEPR** ]   [ **LEP** ]  on the acute feeding induced hypothalamic serotonergic stimulation in normal rats.  Ref: 12873793 Regul PeptRegul Pept, 2003
- At steady states, the absolute increase in plasma leptin  [ **LEPR** ]   [ **LEP** ]  was greater in LP than in EL cows 2.4 ng ml vs 0.4 ng ml.  Ref: 12881203 Am J Physiol Regul Integr Comp PhysiolAm J Physiol Regul Integr Comp Physiol, 2003
- In order to find out whether multiple pregnancy is a factor affecting serum leptin  [ **LEPR** ]   [ **LEP** ]  levels, we compared neonatal serum leptin  [ **LEPR** ]   [ **LEP** ]  concentrations of twins and singleton newborns who were all appropriate for gestational age AGA.  Ref: 12880123 J Pediatr Endocrinol Metab, 2003
- To investigate placental leptin  [ **LEPR** ]   [ **LEP** ]  production in placental insufficiency, placental leptin  [ **LEPR** ]   [ **LEP** ]  production was measured in women with severe preeclampsia group 1 and in normotensive pregnancies associated with intrauterine growth restriction group 2, compared to controls group 3.  Ref: 12865594 Gynecol Obstet Invest, 2003
- These data demonstrate that placental insufficiency is associated with a dramatic increase in placental leptin  [ **LEPR** ]   [ **LEP** ]  production.  Ref: 12865594 Gynecol Obstet Invest, 2003
- CONCLUSION Growth plate chondrocytes possess Ob Rs, and leptin  [ **LEPR** ]   [ **LEP** ]  enhance chondrocyte proliferation and subsequent cell differentiation.  Ref: 12876420 Horm Res, 2003
- Leptin  [ **LEPR** ]   [ **LEP** ]  and obesity .  Ref: 12861270 An Sist Sanit NavarAn Sist Sanit Navar, 2003
- As a consequence of the effects on food intake or energy expenditure, treatment with alphaCRH attenuated the leptin  [ **LEPR** ]   [ **LEP** ]  induced reduction of body adiposity, fat cell size, triglyceride contents, and ob mRNA expression in white adipose tissue.  Ref: 12865337 Endocrinology, 2003
- We investigated the effect of gestational age and maternal nutrition on the maternal plasma concentration of leptin  [ **LEPR** ]   [ **LEP** ]  and cortisol together with effects on fetal adipose tissue deposition plus leptin  [ **LEPR** ]   [ **LEP** ] , IGF I, IGF II ligand, and receptor mRNA abundance near to term.  Ref: 12865340 Endocrinology, 2003
- FRC and blood leptin  [ **LEPR** ]   [ **LEP** ]  were, respectively, negatively and positively correlated with percent body fat.  Ref: 12877823 Pulm Pharmacol Ther, 2003
- There was a significant post surgical decrease in BMI and circulating leptin  [ **LEPR** ]   [ **LEP** ]  levels in all of the treated subjects, but thyroid function did not seem to be affected TSH and fT4 levels were unchanged.  Ref: 12880185 Eat Weight Disord, 2003
- Regulation of leptin  [ **LEPR** ]   [ **LEP** ]  on insulin secretion and sulfonulurea receptor 1 transcription level in isolated rats pancreatic islets.  Ref: 12877797 Chin Med J (Engl), 2003
- The aim of this study was to assess the effect of adding daily exercise to a short term high carbohydrate diet on fasting and postprandial leptin  [ **LEPR** ]   [ **LEP** ]  levels.  Ref: 12861230 Int J Obes Relat Metab DisordInt J Obes Relat Metab Disord, 2003
- Diabetes mellitus and hypertension frequently occur in the same individual, based upon insulin resistance, which is caused by obesity and impairment of secretion of several adipocyte derived hormones adipocytokines, including leptin  [ **LEPR** ]   [ **LEP** ]  and adiponectin.  Ref: 12877076 Nippon Rinsho, 2003
- Selective phosphatidylinositol 3 kinase PI 3 kinase inhibitor wortmannin significantly prevented the leptin  [ **LEPR** ]   [ **LEP** ]  effect on insulin secretion and SUR1 mRNA expression.  Ref: 12877797 Chin Med J (Engl), 2003
- The purpose of this study was to investigate the relationship between the plasma leptin  [ **LEPR** ]   [ **LEP** ]  level and clinical parameters in patients with burn injury.  Ref: 12880724 Burns, 2003
- RESULTS In the presence of leptin  [ **LEPR** ]   [ **LEP** ]  2 nmol L, insulin release was significantly inhibited at either 11.1 or 16.7 mmol L glucose concentration both P <.  Ref: 12877797 Chin Med J (Engl), 2003
- BACKGROUND AND OBJECTIVES Plasma leptin  [ **LEPR** ]   [ **LEP** ]  levels have been shown to be an independent risk factor for cardiovascular disease.  Ref: 12872039 J HypertensJ Hypertens, 2003
- Leptin  [ **LEPR** ]   [ **LEP** ]  has been shown to have sympathetic and vascular effects, and may increase cardiovascular risk through increased blood pressure, left ventricular hypertrophy, or atherosclerotic mechanisms.  Ref: 12872039 J HypertensJ Hypertens, 2003
- We have previously reported that glucose metabolism mediates the effects of insulin to increase leptin  [ **LEPR** ]   [ **LEP** ]  gene expression and leptin  [ **LEPR** ]   [ **LEP** ]  secretion by isolated adipocytes.  Ref: 12878197 Biochem Biophys Res CommunBiochem Biophys Res Commun, 2003
- Effects of leptin  [ **LEPR** ]   [ **LEP** ]  to cultured growth plate chondrocytes.  Ref: 12876420 Horm Res, 2003
- Plasma leptin  [ **LEPR** ]   [ **LEP** ]  is influenced by diet composition and exercise.  Ref: 12861230 Int J Obes Relat Metab DisordInt J Obes Relat Metab Disord, 2003
- The leptin  [ **LEPR** ]   [ **LEP** ]  is a peptide of 167 amino acids, with a signal sequence of 21 amino acids that split up before the leptin  [ **LEPR** ]   [ **LEP** ]  enters the circulatory torrent.  Ref: 12861270 An Sist Sanit NavarAn Sist Sanit Navar, 2003
- In DIO rats on HE diet, leptin  [ **LEPR** ]   [ **LEP** ]  levels increased significantly at 3d followed by increased insulin levels at 7d.  Ref: 12865257 Am J Physiol Endocrinol MetabAm J Physiol Endocrinol Metab, 2003
- Genotype information was collected at 87 loci in a group of 1108 UM HET3 mice bred as the progeny of BALB cJ x C57BL 6J F1 mothers and C3H HeJ x DBA 2J F1 fathers, for which T4, IGF I, and leptin  [ **LEPR** ]   [ **LEP** ]  levels had been measured at 4 and 15 months of age.  Ref: 12865502 Physiol GenomicsPhysiol Genomics, 2003
- For rats fed HF diets made with soybean oil or palm oil, EX also exerted beneficial effects by reducing body fat %, blood insulin, triglyceride and leptin  [ **LEPR** ]   [ **LEP** ]  levels, as well as improving insulin sensitivity.  Ref: 12876304 Exp Biol Med (Maywood), 2003
- Obese children have high leptin  [ **LEPR** ]   [ **LEP** ]  levels, and these may play a role in their earlier onset of puberty.  Ref: 12861226 Int J Obes Relat Metab Disord, 2003
- This involved assessing 3 H thymidine incorporation, alkaline phosphatase ALP activity, proteoglycan production, leptin  [ **LEPR** ]   [ **LEP** ]  receptor Ob R  [ **LEPR** ]  activity, and detection of Ob R  [ **LEPR** ]  using Western blot analysis.  Ref: 12876420 Horm Res, 2003
- There are evidence that increased serotonin metabolism may be involved in leptin  [ **LEPR** ]   [ **LEP** ]  actions.  Ref: 12873793 Regul PeptRegul Pept, 2003
- OBJECTIVE To investigate the regulation of leptin  [ **LEPR** ]   [ **LEP** ]  on insulin secretion and expression of ATP sensitive potassium channel subunit sulfonulurea receptor 1 SUR1 mRNA, and to determine whether the effects of leptin  [ **LEPR** ]   [ **LEP** ]  are mediated through known intracellular signaling transduction.  Ref: 12877797 Chin Med J (Engl), 2003
- To examine the functional role of CRH in the regulation of energy homeostasis by leptin  [ **LEPR** ]   [ **LEP** ] , we measured the effects of the CRH antagonist, alpha helical CRH 8 41 alphaCRH on a number of factors affected by leptin  [ **LEPR** ]   [ **LEP** ]  activity.  Ref: 12865337 Endocrinology, 2003
- The circannual rhythm in SMR could not be explained by changes in body composition, thyroid activity, or leptin  [ **LEPR** ]   [ **LEP** ] .  Ref: 12857676 Am J Physiol Endocrinol Metab, 2003
- Serum level of leptin  [ **LEPR** ]   [ **LEP** ]  was positively correlated with BMI, WHR and percentage of body fat.  Ref: 12880566 Zhonghua Yu Fang Yi Xue Za Zhi, 2003
- The white adipose tissue is the main producer of leptin  [ **LEPR** ]   [ **LEP** ] , acting as a marker of the body s energy reserves.  Ref: 12861270 An Sist Sanit NavarAn Sist Sanit Navar, 2003
- Leptin  [ **LEPR** ]   [ **LEP** ]  intervenes in different physiological processes such as the regulation of the energy balance, the control of appetite and body weight, the metabolism of fats and glucides or reproduction, amongst others.  Ref: 12861270 An Sist Sanit NavarAn Sist Sanit Navar, 2003
- CONCLUSIONS A big difference in serum level of leptin  [ **LEPR** ]   [ **LEP** ]  between obese and normally weighted children was found, suggesting most obese children were resistant to endogenous leptin  [ **LEPR** ]   [ **LEP** ] .  Ref: 12880566 Zhonghua Yu Fang Yi Xue Za Zhi, 2003
- BACKGROUND In humans, serum leptin  [ **LEPR** ]   [ **LEP** ]  correlates positively with fat mass.  Ref: 12864793 Clin Endocrinol (Oxf)Clin Endocrinol (Oxf), 2003
- A search for between strain sequence polymorphisms in the leptin  [ **LEPR** ]   [ **LEP** ]  and NPY genes was unrevealing.  Ref: 12855751 Obes Res, 2003
- While neither fasting nor fasting plus leptin  [ **LEPR** ]   [ **LEP** ]  caused changes in serum prolactin, the increase in prolactin mRNA seen in fasted animals was prevented by leptin  [ **LEPR** ]   [ **LEP** ]  treatment.  Ref: 12856798 Endocr Res, 2003
- CONCLUSIONS The combined IL 12 IL 18 treatment may cause a number of adverse effects independent of TNF alpha and leptin  [ **LEPR** ]   [ **LEP** ]  synthesis.  Ref: 12875675 Chin Med J (Engl), 2003
